# Sex Differences in Microglia Activation in a Rodent Model of Preterm Hypoxic Ischemic Injury with Caffeine Treatment

**DOI:** 10.3390/biomedicines11010185

**Published:** 2023-01-11

**Authors:** Ruth Mae McLeod, Ted S. Rosenkrantz, Roslyn Holly Fitch, Rachel R. Koski

**Affiliations:** 1 Behavioral Neuroscience Division, Department of Psychological Sciences, University of Connecticut, Storrs, CT 06269, USA; 2 Department of Pediatrics, University of Connecticut Health Center and Connecticut Children’s Hospital, Farmington, CT 06030, USA; 3 Division of Neonatology, Department of Pediatrics, University of Minnesota, Minneapolis, MN 55454, USA

**Keywords:** microglia, hypoxia, ischemia, caffeine, preterm

## Abstract

Preterm infants are often treated with caffeine as a respiratory stimulant. However, follow-up data shows caffeine may also have neuroprotective potential. There are several theories as to how caffeine might protect the brain, but none have been proven. This study looked at caffeine effects on microglial activation in rodent brains post hypoxic ischemic (HI) injury. Rat pups underwent either sham or HI surgery on P6, followed by treatment with either caffeine or saline. Forty-eight hours post-injury, brains were collected and underwent paraffin embedding and sectioning followed by immunofluorescence staining. Ionized calcium binding adaptor molecule 1 (Iba-1) was used to label microglia, and 4′,6-diamindino-2-phenylindole (DAPI) was used to label DNA. Cell size measurements of microglia were obtained to gauge microglia activation, and chromatin condensation (DAPI optical density) was used as an index of neuronal cell death. Results suggest that caffeine does offer protective effects, based on significantly increased levels of cell death in HI-saline animals not seen in caffeine-treated HI males and females. However, the mechanism of action may be different. Male HI animals showed marginally reduced microglial activation following caffeine treatment, whereas females did not. Results indicate that though caffeine may act protectively in both sexes by reducing cell death, the benefits may be mediated by different mechanisms.

## 1. Introduction

Approximately 10% of infants in the US are born prematurely, defined as less than 37 weeks gestational age (Center for Disease Control and Prevention, 2021). Premature infants are susceptible to hypoxia ischemia (HI), a form of brain injury that occurs when tissue is deprived of blood and/or oxygen [1]. In the preterm infant, HI is often caused by cardiovascular immaturity that leads to interventricular and/or periventricular hemorrhage (IVH/PVH, defined as bleeding from the capillary-rich germinal matrix into and around the ventricles [1,2]). The resulting oxygen loss leads to white matter injury, though gray matter loss may also be seen in older preterm infants [1,3]. HI can also be caused by reperfusion failure following capillary collapse, due to underdeveloped autoregulation of blood pressure. Both events are associated with periventricular leukomalacia, a non-hemorrhagic white matter injury [1,2]. Chronic lung disease or bronchopulmonary dysplasia in preterm infants can cause hypoxemia (low oxygen), leading to generalized HI in “watershed” regions [1]. Lastly, apnea of prematurity may result in repetitive episodes of oxygen loss with associated brain hypoxia and injury. Early HI injuries are associated with later learning and memory impairments, reduced visuospatial and verbal ability, and frequent motor deficits [4]. This HI injury is very different from HI injuries in term infants.

HI can also occur, though far less frequently, in term infants (>37 weeks GA) with birth complications (e.g., umbilical cord compression, placental dysfunction or abruption). HI in the term infant leads to a form of encephalopathy called hypoxia ischemic encephalopathy (HIE). HIE typically results in greater grey matter damage compared to preterm HI which results in greater white matter damage [1,3]. Infants with HIE (typically >36 weeks GA) are routinely treated with hypothermia, which means decreasing their core head or full body temperature to 33.5 °C for 72 h within the first 6 h of injury. Unfortunately, despite the higher incidence of HI among preterm infants, hypothermia is not approved for this population due to concerns for safety and efficacy, nor is there any other approved protective treatment available [5]. In preterm animal model studies, hypothermia as modeled on term infant therapies has even proven detrimental [6]. However, there are some treatments used for preterm conditions that have shown potential neuroprotective effects. One such drug is caffeine, which is used to treat apnea of prematurity. In longitudinal studies, caffeine has been shown to improve cognitive outcomes [7,8,9,10,11,12]. However, the mechanism of protection is unknown.

Caffeine is a non-specific adenosine antagonist, blocking action at all adenosine receptors. Caffeine also reduces plasma inflammatory markers [13]. A single caffeine dose was found to improve open field and beam walking (motor assessment) outcomes and reduce lymphocytes 24 h post-injury in a mouse model of neonatal HI [14]. Interestingly, the immunomodulatory effect was seen with caffeine but not hypothermia, suggesting a different mechanism of neuroprotective action [14]. Caffeine treatment immediately after HI in a postnatal day 10 (P10) mouse model was also found to reduce brain injury, as well as activated microglia, apoptotic cell activity, and pro-inflammatory cytokines [15]. In neonatal rat studies, post-HI treatment with caffeine citrate was found to rescue several behaviors that are typically impaired following induced injury [6,16], as well as enhancing neurogenesis [17] and attenuating cell death and inflammation [18,19,20,21].

Most theories of caffeine protection focus on the A1 or A2A receptor (A1R, A2AR). Although under normal conditions the A1R prevents excess calcium influx, studies suggest in conditions of high adenosine (as seen with HI brain injury), this receptor may alter its function and begin to trigger *more* calcium influx—an effect associated with mitochondrial dysfunction and cell death [22,23]. Thus, under conditions of HI injury, A1R antagonism could be protective—specifically by preventing excess calcium influx and reducing neuronal cell death [24]. However, evidence of protection at the A1R is mixed. In a recent review it was noted that agonism of A1R can increase inflammation (though not cell death) or reduce it in other cases [25]. In THP-1 macrophages, A1R activation was found to increase proinflammatory factors Interleukin (IL)-6 and IL-1b. On the other hand, A1 is also involved in inflammation resolution and tissue repair [26]. Finally, while some studies show A1R antagonism to be protective, others report that agonism is beneficial [27].

The A2A receptor (A2AR) is another potential site of caffeine protection. On neurons, A2A antagonism can reduce excitotoxicity by blocking calcium influx, but does so less effectively than via A1 antagonism [28]. On microglia, it is hypothesized that antagonism of A2AR may prevent overactivation, therefore reducing inflammation and cell death [29]. Some studies specifically indicate that A2A antagonism decreases inflammation and provides neuroprotection via microglia during post-injury in caffeine-treated subjects for a variety of neurodegenerative diseases [30]. A2AR antagonism also reduces inflammation in animals treated with a low protein diet, whereas agonism of microglial A2AR was found to increase inflammation [29]. Given the robust increase in the presence and activation of microglia in the brain following HI, it is possible that microglial A2AR directly mediates caffeine-based protection and anti-inflammatory effects [31].

The goal of the current study was to test the impact of post-HI caffeine treatment on microglial activation in a rat preterm HI model on postnatal day 6 (P6; an age considered roughly equivalent to a GA 26–30 human infant [32]). We hypothesized decreased cell death in rats that received caffeine post HI injury as compared to those treated with saline. We also hypothesized that levels of activated microglia would be increased in animals with injury, but less so following caffeine treatment. Finally, we predicted that this study would show sex differences in HI and caffeine-treated HI subjects. In general, after prematurity, females have better outcomes than males [33,34,35,36,37,38]. Moreover, sex differences can occur in response to treatment. For example, female rats with neonatal HI showed increased protection and improved outcomes with hypothermia treatment as compared to males [39,40]. In the current study, we predicted that females with HI might show less cell death than HI males, and possibly less post-injury microglial activation. We were unsure of how reduced microglial activation in HI animals treated with caffeine might interact with sex.

## 2. Materials and Methods

### 2.1. Subjects

**Figure 1:**
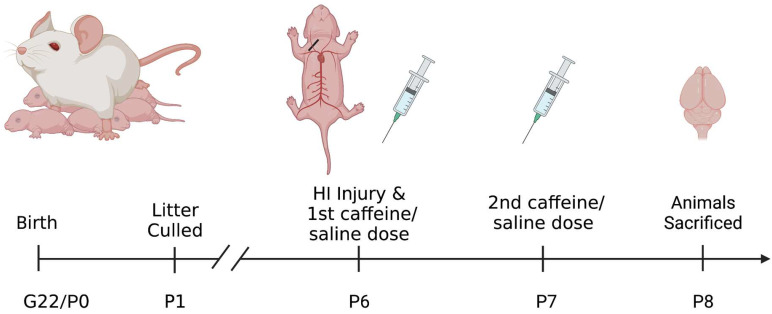
Diagram of the experimental design shown in a timeline from subject birth to sacrifice. Following sacrifice brains were collected and sectioned for staining. Created with BioRender.com.

Subjects were Wistar pups (male and female) born to time-mated dams. Dams were shipped to the University of Connecticut after mating (E5) and were maintained in single housing tubs at our AALAC-accredited animal facility. The day after pups were born (P1), litters were culled and fostered to a total of 8 pups (4 male, 4 female) (Figure 1).

### 2.2. HI Induction

On P6, pups were assigned to one of 4 treatment groups: Sham with saline treatment; Sham with caffeine treatment; HI with saline treatment; or HI with caffeine treatment. The sham animals act as controls for this experiment. All four treatment groups were balanced within litters, with one male and one female from each group (total eight pups per litter). HI was then induced following the Rice–Vannucci method [41]. Sham control animals were placed under anesthesia with isoflurane and a small longitudinal incision was made to the right of the midline in the neck, on the ventral side. The right common carotid artery was located and separated from the tissues surrounding it but was not cauterized, followed by suturing of the wound. HI pups underwent the same anesthesia and surgery except that the carotid artery was cauterized. Again, the incision was sutured, and all pups were given a small subcutaneous injection of bupivacaine for analgesia. Pups received a foot tattoo for identification and were allowed to recover from anesthesia in an incubator, followed by return to their dams to feed. About 120 min later (after nursing), animals were placed in warmed chambers to maintain body temperature (~98 F) for 90 min. HI subjects were placed in a closed hypoxia chamber with 8% oxygen (balanced with nitrogen) and Sham control subjects were placed in open chambers in room air (21% oxygen).

### 2.3. Caffeine Treatment

On removal from the chambers, animals were given an intraperitoneal (i.p.) injection of either caffeine citrate (25 mg/kg using individual pup weights) or an equivalent amount of vehicle (sterile physiological saline). This amount was chosen because it is the same dosage used clinically for apnea in preterm infants. Pups were returned to their dams and given a second caffeine or saline injection 24 h post HI injury (receiving a total of 2 doses). This resulted in the following groups, Sham Saline Male = 6, Sham Saline Female = 3, Sham Caffeine Male = 6, Sham Caffeine Female = 3, HI Saline Male = 11, HI Saline Female = 7, HI Caffeine Male = 12, HI Caffeine Female = 8. Later there was no difference found between the Sham Saline and Sham Caffeine groups (*p* > 0.1), so these groups were combined. This resulted in Sham Male = 12 and Sham Female = 6.

### 2.4. IBA-1 and DAPI Staining

Animals were perfused under anesthesia (Isoflurane) at 48 h post-HI injury (P8) (Figure 1) using 4% paraformaldehyde in phosphate buffer solution as a fixative. Brains were paraffin embedded, cryo-sectioned coronally (60 µm) and every section was saved from approximately the start of the hippocampus up to the cerebellum (on average about 24 sections per subject). Mounted sections were stained using 4′,6-diamidino-2-phenylindole (DAPI; 1:500 dilution, Thermo Fisher, Waltham, MA, USA), and ionized with calcium binding adaptor molecule 1 antibody (IBA-1, which binds specifically to microglia; 1:1000 dilution, Wako). Sections were blocked in normal goat serum and incubated in primary antibody-(Iba-1), followed by incubation with appropriate secondary antibody, anti-rabbit Alexa flora 488 (Thermo Fisher, Waltham, MA, USA). Sections were cover-slipped with Fluromount G mounting media (Southern Biotech, Birmingham, AL, USA) and sealed.

**Figure 2:**
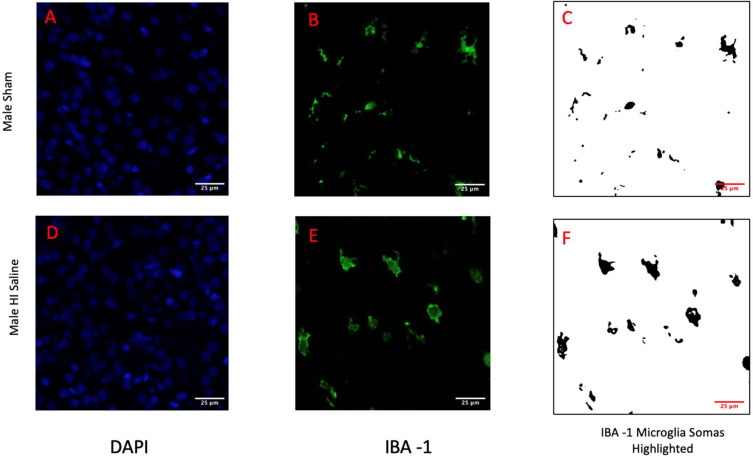
Displayed are the 80× images collected from the right cortex for both a male sham subject (**A**–**C**) and a male HI subject (**D**–**F**). DAPI images (**A**,**D**) highlight the chromatin of the cells and the condensation of that chromatin indicates cell death. IBA-1 images (**B**,**C**,**E**,**F**) highlight microglia. Images (**C**,**F**) show the selection of the microglia soma from the images. The area of these cell somas are used to indicate activation.

Since DAPI labels nuclear chromatin, focal concentration of DAPI stain was used as an index for chromatin condensation indicative of apoptotic cell death. Since IBA-1 labels the cell membrane of microglia, we used this label to measure soma size and, indirectly, microglial activation (since activated microglia have a larger soma than homeostatic microglia). Importantly, measures of microglial soma size have been shown to index activation as accurately as high-magnification cell tracings [42,43]. To obtain these measures, images (80×) were captured from the right cortex (right = side of injury) using a *Nikon AR1* Confocal florescent microscope. A blue fluorescent filter was used to capture images for DAPI, and a green florescent filter for IBA-1 (yielding two image-sets for each subject). *ImageJ* was used to assess optical distribution of the DAPI (blue), with more concentrated (lower) DAPI optical distribution indicating higher chromatin condensation (more cell death). Microglia soma cell size was measured using *ImageJ* on 80× images (green), with a larger mean soma size indicating heightened microglial activation (Figure 2). Approximately 5 serial images were measured from the right cortex per subject, following anatomic criteria defined in *Atlas of the Neonatal Rat Brain* [44]. All image quantification was performed blind to subject identification and treatment.

No behavioral testing was able to be completed in this study due to sacrificing the animals at P8 (48 h post-HI injury) to capture the activation of the microglia cells. Previous animal models have shown microglia cells are activated between approximately 6–72 h (more profound at 48 h) in lipopolysaccharide challenged rodent models which induce systemic and neuroinflammation with microglia cells returning to the resting state at about 7 days [45]. However, previous animal studies using this similar HI animal model have demonstrated caffeine neuroprotection with improved behavioral outcomes in spatial learning and memory testing [6,16].

### 2.5. Analysis

After collection of measurements from DAPI images (optical distribution) and Iba-1 images (microglial soma size), values from Sham saline and Sham caffeine control groups were statistically compared (*p* > 0.1). Since no differences were found on any measure, the Sham control groups were combined. Next, data were analyzed using the between-variables of Group and Sex (multi-variate ANOVAs). Tukey Tests were used to compare specific groups following *a priori* planned comparisons. Where appropriate given specific one-directional *a priori* hypotheses and/or prior data, one-tailed tests were used. Male and female data were analyzed by using Sex as a between-variable, or were analyzed separately. Final n at sacrifice was 59. Some samples were not utilized due to poor quality of sectioning or staining (noting some samples yielded quality images for one stain but not the other). Final n’s were: DAPI, 56 (Sham M = 12, Sham F = 6, HI M = 11, HI F = 7, HI Caf M = 12, HI Caf F = 8); Iba-1, 50 (Sham M = 10, Sham F = 6, HI M = 10, HI F = 5, HI Caf M = 12, HI Caf F = 7).

## 3. Results

### 3.1. DAPI

**Figure 3:**
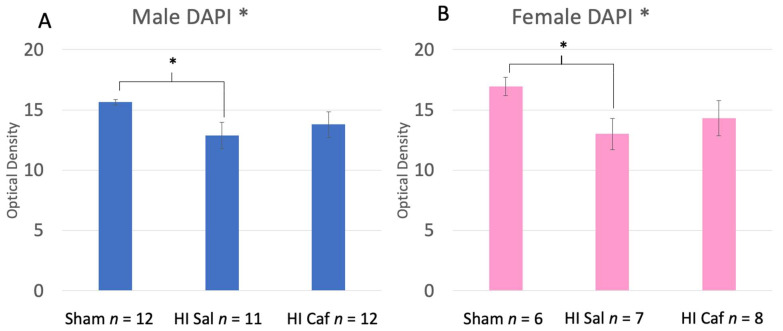
These two graphs display the DAPI results of the male (**A**) and female (**B**) animals. Sham refers to the animals that did not receive the HI procedure, HI Sal refers to animals that received the HI procedure but only received a saline treatment, and HI Caf refers to animals that received the HI procedure and were treated with caffeine. DAPI stain was used to measure chromatin condensation through optical density. The lower the optical density, the greater chromatin condensation and thus cell death (lower optical density indicates higher cell death). There was no Sex difference seen between Groups (*p* = 0.456 but displayed separately to illustrate comparable protection). There was an overall Group difference (*p* = 0.012), and based on a Tukey post-hoc analysis, a difference between HI saline and Sham animals (*p* = 0.012) was seen. Though there is no significant difference between HI saline and HI caffeine animals, the lack of difference between HI caffeine and Sham animals supports a partial reduction in cell death in caffeine-treated animals at 48 h post injury. * indicates a *p*-value less than 0.05.

No Sex difference was seen overall (F(1,56) = 0.542, *p* = 0.465), nor an interaction between Sex and Group (F(2,56) = 0.153, *p* = 0.853). Therefore, data for this measure were combined and analyzed across males and females (note that in Figure 2, male (A) and female (B) values are presented separately for illustration). An overall Group effect was observed (F(2,56) = 4.828, *p* = 0.012), with Post-hoc Tukey tests showing a significant difference between the combined Sham control and HI saline groups (*p* = 0.012; Figure 2; lower optical distribution values in HI saline groups reflects increased chromatin condensation). There was no difference between HI saline and HI caffeine animals (*p* = 0.556), nor between Sham control and HI caffeine animals (*p* = 0.115; Figure 3).

### 3.2. IBA-1

For IBA-1, we found Sex by Group differences, including a significant interaction between male and female Sham control vs. HI saline groups (F(1,31) = 3.332, *p* = 0.04), as well as a marginal one-tailed interaction between male and female HI saline vs. HI caffeine groups (F(1,34) = 1.653, *p*= 0.1). We thus performed further IBA-1 analyses within each Sex separately.

**Figure 4:**
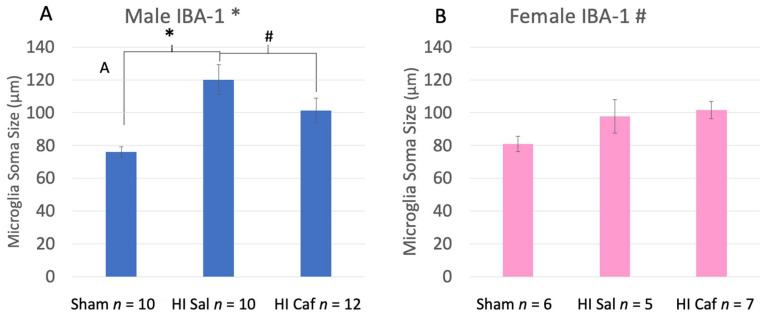
This figure displays male (**A**) and female (**B**) IBA-1 microglia soma size measurements side by side. Sham refers to the animals that did not receive the HI procedure, HI Sal refers to animals that received the HI procedure but only received a saline treatment, and HI Caf refers to animals that received the HI procedure and were treated with caffeine. For male animals there was an overall Group difference (*p* = 0.001). HI saline animals had a significant increase in microglia soma area, indicating more activation than Sham animals (*p* = 0.01). There was also a marginal one-tailed difference between HI saline and HI caffeine male animals (*p* = 0.08), indicating a reduction in activation after caffeine treatment. For female animals there was a marginal overall effect (*p* = 0.09), but no difference between HI saline and Sham animals nor HI saline and HI Caffeine animals. This indicates that caffeine failed to reduce activation in caffeine-treated HI females. * indicates a p value less than 0.05, # indicates a p value less than 0.1 but greater than 0.05.

Male data revealed a significant overall Group effect (F(2,32) = 9.225, *p* = 0.001). On post-hoc analysis we found a significant increase in microglia activation specifically in HI saline animals compared to Sham controls (*p* = 0.01), reflecting larger mean soma sizes. In addition, we saw a marginal difference between HI saline and HI caffeine animals (*p* = 0.08, one-tail; Figure 4A), with less activation in caffeine-treated HI males.

Female data showed a marginal overall Group effect (F(2,18) = 2.884, *p* = 0.09). However, females did *not* show the expected difference between HI saline and Sham control animals as was seen in males (i.e., no increase was observed in microglial activation for HI saline subjects). There were also no post-hoc differences between HI saline and Sham control animals (*p* = 0.238), nor between HI saline and caffeine animals (*p* = 0.907; Figure 4B).

## 4. Discussion

The current results confirm that P6 HI in rats leads to significantly increased apoptotic cell death in the cortex at 48 h post-HI injury, as measured by chromatin condensation compared to sham control animals. Although caffeine did appear to decrease damage, cell death values were not lowered to Sham control levels (Figure 3). In fact, results showed that the HI caffeine group was not significantly different from *either* Sham control or HI saline values. This result is consistent with related evidence of partial but not complete recovery of neuroanatomy and behavior outcomes following caffeine treatment [6,16].

IBA-1 results confirmed a significant activation of microglia in male rats with P6 HI (larger mean soma size). Results also showed a partial protection for male animals following caffeine treatment, based on the marginal reduction of mean microglial soma size. Females, on the other hand, did not show the same pattern. Both female HI groups showed non-significant increased microglial activation compared to the female Sham (Figure 4B). These results suggest an attenuated inflammatory/microglial response to HI injury in neonatal females as compared to males, as measured in the cortex. Moreover, caffeine does not appear to impact microglial activation in HI injured females. Nonetheless, overall results did show similar increased cell death in both sexes with HI injury, as well as comparable protective effects of caffeine as reflected in reduced cell death. This is consistent with other evidence of improved behavioral outcomes following caffeine treatment in neonatal HI rodent models of both sexes [6,16]. However, the current microglial results suggest that the similar protective effects of caffeine on cell death in males and females appear to occur through different protective pathways.

This sex difference is very interesting, but not entirely unexpected. Maturation of microglia is delayed in males, and microglia are more sensitive to inflammation in neonatal males [46]. Male microglia are more mobile, while female microglia show higher expression of cellular repair and inflammatory control genes and are generally more protective [47]. These microglia differences are likely due to several developmental factors. First, the paternal X chromosome is largely silenced in female immune development but is escaped by 15% of paternal immune genes. This does not occur on the Y chromosome (with only one X). This sex disparity leads to more active immune gene transcription in females [47]. Second, microglia are sensitive to estrogen and testosterone, which may modify function in a sex-differentiated fashion based on elevated circulating testosterone levels in fetal and newborn males [48,49]. Testosterone may specifically be implicated, since ovarian hormone depletion in older females changed microglia activation patterns but not in young females [47]. Finally, in A2A depleted mice, microglial sex differences have been reported in the prefrontal cortex and dorsal hippocampus, with females showing more complex microglial activation in the prefrontal cortex, and males in the dorsal hippocampus [50]. Males also have more microglia in some brain areas (hippocampus and cortex), and higher antigen presenting capacity [51]. These factors may explain in part the sex difference in patterns of microglial response observed.

Limitations to the scope of this study were present. The constraint of subject numbers meant that we could not match littermates with the same treatment type who would not be sacrificed at P8. This would have allowed us to not only look at microglial effects at P8 but compare the behavior of the matched littermates rather than relying on the results of other caffeine studies. Future studies should look at getting both microglial measures and behavior. In addition, this study focused on one timepoint (48 h post-HI injury). Ideally, future studies could focus on additional timepoints to further measure the progression of microglial activation after caffeine administration.

Since it appears that there are multiple protective pathways mediating neuroprotection from caffeine, as well as sex differences in the preferential activation of these different pathways (much as is seen in apoptotic cell death cascades) [52,53,54], it will be very important for future neuroprotective studies to explore these factors. This includes further exploration of the adenosine receptor target mechanism, the mixed evidence regarding inflammatory effects of the A1 receptor, and caffeine’s effect on whole body inflammation through the A2B receptor. The latter has not been a common focus in caffeine inflammation studies, but A2B has a pro-inflammatory role in most cells (epithelial cells, smooth muscle, and fibroblasts), and it limits endothelial cell inflammation and prevents macrophage activation [55]. A2B receptors also help regulate inflammation and programed cell death [26]. As mentioned in the introduction, A1 antagonism could be protective by preventing excess calcium influx and reducing neuronal cell death [24]. A1 is also involved in inflammation resolution and tissue repair [26]. Given mixed evidence, specific studies using targeted receptor antagonists to dissociate caffeine’s binding mechanisms will be important to interpreting protective effects and refining next-generation targeted therapeutics for preterm infants.

## Data Availability

Not applicable.
